# A One-Dimensional Hemodynamic Model of the Coronary Arterial Tree

**DOI:** 10.3389/fphys.2019.00853

**Published:** 2019-07-09

**Authors:** Zheng Duanmu, Weiwei Chen, Hao Gao, Xilan Yang, Xiaoyu Luo, Nicholas A. Hill

**Affiliations:** ^1^Key Laboratory of the Ministry of Education for Optoelectronic Measurement Technology and Instrument, Beijing Information Science and Technology University, Beijing, China; ^2^Guangxi Key Laboratory of Regenerative Medicine, Research Centre for Regenerative Medicine, Guangxi Medical University, Guangxi, China; ^3^School of Mathematics and Statistics, University of Glasgow, Glasgow, United Kingdom; ^4^Second Affiliated Hospital of Nanjing Medical University, Nanjing, China

**Keywords:** hemodynamics, coronary arteries, blood flow, pressure, wave intensity, vascular rarefaction, structured tree model

## Abstract

One-dimensional (1D) hemodynamic models of arteries have increasingly been applied to coronary circulation. In this study, we have adopted flow and pressure profiles in Olufsen's 1D structured tree as coronary boundary conditions, with terminals coupled to the dynamic pressure feedback resulting from the intra-myocardial stress because of ventricular contraction. We model a trifurcation structure of the example coronary tree as two adjacent bifurcations. The estimated results of blood pressure and flow rate from our simulation agree well with the clinical measurements and published data. Furthermore, the 1D model enables us to use wave intensity analysis to simulate blood flow in the developed coronary model. Six characteristic waves are observed in both left and right coronary flows, though the waves' magnitudes differ from each other. We study the effects of arterial wall stiffness on coronary blood flow in the left circumflex artery (LCX). Different diseased cases indicate that distinct pathological reactions of the cardiovascular system can be better distinguished through Wave Intensity analysis, which shows agreement with clinical observations. Finally, the feedback pressure in terminal vessels and measurement deviation are also investigated by changing parameters in the LCX. We find that larger feedback pressure increases the backward wave and decreases the forward one. Although simplified, this 1D model provides new insight into coronary hemodynamics in healthy and diseased conditions. We believe that this approach offers reference resources for studies on coronary circulation disease diagnosis, treatment and simulation.

## 1. Introduction

With the ever increasing load of coronary heart disease, studying the coronary arteries is of importance. Coronary blood flow and pressure are key components in cardiac pump function. Clinical measurements, particularly non-invasive ones, are difficult to perform and necessarily limited in terms of resolution (Toyota et al., 2005; Chiribiri et al., 2013). Invasive dynamic measurements are regularly performed in the coronary circulation system, and in many cases may be clinically indicated, but they do carry some risk, so non-invasive approaches would be preferable in circumstances where invasive measures are not indicated. Mathematical modeling of coronary vessels is an alternative approach to understanding the relationship between coronary flow/pressure and physical properties of the cardiovascular system, at required locations, and it also provides useful insights, such as feedback control of the coronary vascular resistance and wave intensity.

Over the last twenty years, various mathematical models have been developed for cardiovascular problems, ranging from one segment of the vessel to the whole vessel system (Pietrabissa et al., 1996; Kim et al., 2010; Mynard, 2011; Karimi et al., 2013). Although most of the models are developed for systemic circulations, the mathematical principles are similar for coronary circulations. The simplest approach is the so called lumped-parameter or zero-dimensional (0D) modeling. This is based on a resistance-compliance Windkessel approach for the circulation system (Frank, 1899; Pietrabissa et al., 1996). This type of model has been used to simulate the cardiovascular system and the distribution of the blood flow and as an investigative tool to evaluate the different bypass treatments (Pietrabissa et al., 1996). Zero-dimensional models are also widely adopted as out-flow boundary conditions to provide a physiologically accurate pressure or flow profile (Huo and Kassab, 2007; Alastruey et al., 2008; Mynard, 2011). It is particularly useful for evaluating the effects of micro-circulation in patient-specific simulations (Alastruey et al., 2008). Many studies have also demonstrated that the myocardial contraction provides extra forces to the terminal coronary wall (Spaan et al., 2000; Algranati et al., 2010). Mynard (2011) established a comprehensive hemodynamic model for coronary arteries in which a 0D model was used at the boundary to reflect the effects of micro-vascular beds. Duanmu et al. (2018) recently developed a patient-specific 0D model of coronary circulation in which the root impedance was evaluated based on a static structured tree model, and good agreement with experiments was obtained.

The major limitation of the 0D approach is over-simplification in omitting the wave information. In addition, very few studies have considered the interaction between the ventricular contraction and the coronary arteries when modeling the coronary flow (Van der Horst et al., 2013; Mynard et al., 2014; Mynard and Smolich, 2015). This is because 0D models are unable to simulate the wave effects due to heart fluctuations (Van der Horst et al., 2013). Most of the coronary models use 0D terminal boundary conditions as feedback from ventricular contraction (Mynard et al., 2014; Mynard and Smolich, 2015), which is a crude approximation for the physiological vascular bed.

In one-dimensional (1D) models, the coronary arteries are treated as tapered tubes. The governing equations for the flow are derived from the axisymmetric Navier–Stokes equations, and the vessel walls are assumed to be either rigid or compliant (Olufsen, 1999). Owing to its simplicity and a yet more rational description of the physiology, 1D modeling has been widely used in circulation systems, such as the systemic arteries (Olufsen, 1999; Olufsen et al., 2000), microcirculation (Alastruey et al., 2008) and coupled models Huo and Kassab (2007); Mynard (2011). Olufsen et al. studied such a model for systemic circulation in which a structured tree boundary condition was used (Olufsen, 1999; Olufsen et al., 2000). A 1D model was also developed for subject-specific human pulmonary circulation (Qureshi and Hill, 2015). Similar approaches were used to predict flow and pressure in the pulmonary arteries (Olufsen et al., 2012), which showed that a decrease of the compliance in the large arteries leads to a pressure rise. Stergiopulos et al. (1992) showed that a 1D model can provide a better result than the 0D model in accounting for the nonlinear properties of the arterial wall. 1D modeling is often used to build a coupled circulation system with frequency outlet condition. Recently, Chen et al. (2016) built a coupled system between a three-dimensional finite-strain left ventricle and a physiologically-based 1D model of systemic circulation, and studied the effects of rarefaction and arterial stiffness on the performance of the heart.

With the development of medical imaging technologies, detailed 3D coronary arterial models can now be reconstructed from *in vivo* imaging (Mittal et al., 2005; Raff et al., 2009). Simulations of 3D circulation systems have been performed with different outlet boundary conditions (Vignon-Clementel et al., 2006; Kim et al., 2010). Such an approach, however, is extremely time consuming and the accuracy of the result depends on the parametric boundary conditions, which are usually difficult to obtain. For this reason it is very important to continue to develop more accurate patient-specific 1D models for the coronary system, in order to provide a real-time clinical support system.

One of the advantages of 1D modeling is to be able to analyze wave intensity (WI), which is important in evaluating the myocardial and hemodynamic functions. The waveform of the pressure or flow in the arteries can provide a convenient way to assess the wave energy transmission in a time domain-based manner in the cardiovascular system (Parker, 2009). In addition, biventricular pacing that increases the coronary blood flow velocity from diastolic-dominant backward decompression waves can be detected though WI analysis (Kyriacou et al., 2012). Combined analysis of pressure and flow velocity using WI analysis demonstrates that coronary hemodynamics are naturally most reflective of the upstream fluid dynamics of a stenosis Sen (2013). However, WI analysis based on measured flow and pressure alone can sometimes be inaccurate or even misleading (Sun et al., 2000; Sen et al., 2014; Mynard and Smolich, 2015) due to the difficulties in measuring coronary flow and pressure *in vivo*. When used in a robust computational model, WIA can quantify contributions of pressure and flow waveforms at arbitrary locations and different physiological conditions (Willemet and Alastruey, 2015).

In this paper, we have developed a 1D model of the large coronary arteries based on *in vivo* CT scans. This model takes account of the detailed coronary geometry and the pulsatile pressure loading within the myocardium. Unlike previous coronary models with boundary conditions estimated from 0D models (Mynard et al., 2014; Mynard and Smolich, 2015), we use a structured-tree model (Olufsen, 1999; Olufsen et al., 2000) for the vascular beds of smaller coronary vessels to provide the boundary conditions relating pressure and flow at the distal end of each terminal artery, added to an additional pressure feedback term resulting from the ventricular wall contraction. We perform WI analysis of the left circumflex artery under different pathological conditions, such as increased arterial wall stiffness and vascular rarefaction in the structured trees. Finally, the effects of the feedback pressure and geometry uncertainties are investigated.

## 2. Methods

### 2.1. Reconstruction of the Coronary Vessels

The right and left coronary trees are reconstructed from the *in vivo* data from a male patient (age 61, 80 kg). GE 16-slice CT angiography was performed and medical imaging software (GE, AW) was used for visualization and measuring vessel length and radius. Blood flow data for the left circumflex branch of the coronary vessels was measured by an intra-coronary Doppler guide wire (FloWire, 1400-Floppy). The measurements were performed by The Second Affiliated Hospital of Nanjing Medical University, China. All the procedures were conducted according to established ethical guidelines (Raff et al., 2009). This study was approved by the Human Experiment Committee of The Second Affiliated Hospital of Nanjing Medical University. The patient provided written consent.

**Figure 1:**
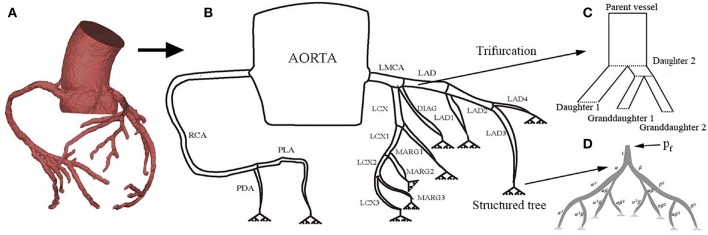
The structure of the coronary tree, including the right and left coronary arteries Raff et al. (2009): **(A)** CT reconstruction of coronary circulation, **(B)** the 1D coronary tree model, **(C)** modeling of the trifurcation, and **(D)** the terminal branches are connected to a capillary bed in the myocardium using a structured tree model with feedback pressure *p*_*f*_(*t*).

In order to reconstruct the coronary tree structure, each vessel was rebuilt along the center line from the CT angiography data at diastole during maximum hyperemia. The coronary tree is separated into segments according to the clinical anatomy following the American Heart Association Standard (Raff et al., 2009). The coronary trees are separated into two branches: the left main coronary artery (LMCA), which includes the left anterior descending branch (LAD), the left circumflex branch (LCX), the diagonal artery (DIAG), and the marginal branch (MARG), and the right coronary artery (RCA) which includes the posterior descending artery (PDA) and the posterior lateral artery (PLA). In the left branch, each segment is further divided into several segments as shown in Figure 1. Each vessel is tapered from the proximal to distal ends. The measured geometries of all the vessels are listed in Table 1.

**Table 1:** Geometries (± 0.1 mm) of the coronary tree measured from the CT images.

**Branch**	**Length (mm)**	**Proximal Radius top (mm)**	**Distal Radius bottom (mm)**
RCA	109	1.51	1.48
PDA	37	1.08	1.05
PLA	42	1.28	1.25
LMCA	32	1.88	1.79
LAD	27	1.51	1.45
LAD1	29	1.46	1.45
LAD2	69	1.38	1.06
LAD3	47	1.03	1.03
LAD4	22	0.88	0.77
DIAG	35	1.26	1.16
LCX	21	1.48	1.47
LCX1	16	1.32	1.29
LCX2	20	1.15	1.14
LCX3	39	1.02	0.99
MARG1	35	1.16	1.08
MARG2	30	0.98	0.96
MARG3	37	1.02	1.00

Following Olufsen (1999) and Olufsen et al. (2000), the distal end of each terminal large artery is connected to a vascular bed (represented in the model by a structured tree) of smaller vessels that lie within the myocardium. In the structured tree, each parent vessel of radius *r*_*p*_ is divided into two daughter arteries with radii *r*_*d*_1__ = α*r*_*p*_ and *r*_*d*_2__ = β*r*_*p*_, where 0 < β < α <1. The branching process continues until the radii of daughter vessels reach the minimum radius, *r*_*m*_. The bifurcation process obeys:

(1) $$\begin{array}{l} r_{p}^{\xi }=r_{d_{1}}^{\xi }+r_{d_{2}}^{\xi },\;2.33\leq \xi \leq 3, \end{array}$$

(2) $$\begin{array}{l} \gamma ={\left(r_{d_{1}}/r_{d_{2}}\right)}^{2},\;\eta =\frac{r_{d_{1}}^{2}+r_{d_{2}}^{2}}{r_{p}^{2}},\;\eta =\frac{1+\gamma }{{\left(1+\gamma^{\xi /2}\right)}^{2/\xi }}. \end{array}$$

where *ξ* is the radius exponent, *η* is the area ratio, and γ is the asymmetry ratio. It should be noted that the parameters are related.

### 2.2. 1D Coronary Model

In the 1D model, we consider the vessel wall to be a deformable tapering tube, whose radius and wall properties are functions of the centerline distance *x* from the proximal end of the vessel. We further assume the vessel wall is impermeable, and that blood is an incompressible Newtonian fluid. The continuity and momentum equations are:

(3) $$\begin{array}{ll} \frac{\partial A}{\partial t}+\frac{\partial q}{\partial x} & =0, \end{array}$$

(4) $$\begin{array}{ll} \frac{\partial q}{\partial t}+\frac{\partial }{\partial x}\frac{q^{2}}{A}+\frac{A}{\rho }\frac{\partial p}{\partial x} & =-\frac{2r\pi \mu q}{\delta A}, \end{array}$$

where *A* is the cross-sectional area, *q* = *uA* is the flow rate, *u* is the velocity, ρ is the blood density, μ is the blood viscosity, *r*(*x, t*) is the vessel radius, and δ is the thickness of the boundary layer (Olufsen, 1999; Olufsen et al., 2000). The blood pressure *p* is related to *A* in the tube-law:

(5) $$\begin{array}{l} p-p_{0}=\frac{4Eh}{3r_{0}\left(x\right)}\left(1-\sqrt{\frac{A}{A_{0}}}\right), \end{array}$$

where *r*_0_ is the vessel radius, *h* is the wall thickness, *p*_0_ is the reference pressure, and *A*_0_ is the cross sectional area when *p* = *p*_0_, and *E* is the Young's modulus, which is a function of *r*_0_ given by:

(6) $$\begin{array}{l} \frac{Eh}{r_{0}}=k_{1}e^{k_{2}r_{0}}+k_{3}, \end{array}$$

where the coefficients *k*_1_, *k*_2_, *k*_3_ are taken from Olufsen (1999). These equations are solved numerically using a Lax–Wendroff method (Lax and Wendroff, 1960).

Unlike the structured tree models for systemic and pulmonary circulations, where only bifurcations exist, a trifurcation is observed in the coronary circulation at the end of LMCA (consisting of LCX, LAD and DIAG) as shown in Figure 1C. To use the same branching process (Equations 1, 2), we divide the trifurcation into two bifurcations. Specifically, the LMCA is separated into daughter 1 (the DIAG) and daughter 2 with a radius of 0.185 cm derived from (1). The daughter 2 is further divided into two granddaughter vessels: LCA and LCX. The length of daughter 2 is small (0.25 cm) so that the structure is close to the original trifurcation.

### 2.3. Impedance of the Vascular Beds

The structured-tree model is used as the outflow boundary of each *terminal* large vessel as shown in Figure 1D. In the frequency domain, the linearized momentum and continuity equations for the small arteries in the terminal vascular bed are (Olufsen, 1999):

(7) $$\begin{array}{l} i\omega Q+K\frac{A_{0}}{\rho }\frac{\partial P}{\partial x}=0, \end{array}$$

(8) $$\begin{array}{l} i\omega CP+\frac{\partial Q}{\partial x}=0, \end{array}$$

where ω is the frequency and *K* is a parameter related to the Womersley number (Olufsen, 1999), $C≃\frac{3A_{0}r_{0}}{2Eh}$ is the vessel compliance, and *P*(*x*, ω), *Q*(*x*, ω) are the complex Fourier transforms of *p*(*x, t*) and *q*(*x, t*), and ω takes the values that are integer multiples of the cardiac frequency.

From (7) and (8) we obtain:

(9) $$\begin{array}{l} \frac{\omega^{2}}{c^{2}}Q+\frac{\partial^{2}Q}{\partial x^{2}}=0\;\text{and }\frac{\omega^{2}}{c^{2}}P+\frac{\partial^{2}P}{\partial x^{2}}=0, \end{array}$$

where *c* is the wave propagation velocity in the small vessels of the structured-tree,

(10) $$\begin{array}{l} c=\sqrt{\frac{A_{0}K}{\rho C}}. \end{array}$$

The impedance *Z*(*x*, ω) is computed from:

(11) $$\begin{array}{l} P\left(x,\omega \right)=Z\left(x,\omega \right)Q\left(x,\omega \right). \end{array}$$

At a vessel bifurcation, conservation of flow and continuity in pressure lead to:

(12) $$\begin{array}{l} \frac{1}{Z_{p}}=\frac{1}{Z_{d_{1}}}+\frac{1}{Z_{d_{2}}}, \end{array}$$

where *Z*_*p*_ is the impedance of the parent vessel, and *Z*_*d*_1__ and *Z*_*d*_2__ are the impedances of the daughter vessels of diameters *d*_1_ and *d*_2_. Using (9) and (12), we can deduce the root impedance for a vessel of length *l*:

(13) $$Z(0,\omega )=-\frac{ig\sin(\omega l/c)+Z(l,\omega )\cos(\omega l/c)}{\cos(\omega l/c)+igZ(l,\omega )\sin(\omega l/c))},$$

where *Z*(*l*, ω) is the impedance at the distal end of the vessel, and $g=\sqrt{CA_{0}K/\rho }$. Thus, (9), (12) and (13) can be solved recursively to obtain the root impedance of the terminal vessel of the vascular beds (Olufsen et al., 2000).

### 2.4. Inlet and Boundary Conditions

For a given ventricular pressure, the inlet flow rates to the left and right coronary branches are estimated from a lumped parameter model, for a heart rate of *t*_*c*_ = 0.9*s* (Pietrabissa et al., 1996). The coronary blood flow is 4–5% of the cardiac output (Hall, 2015). We determine the left ventricular and right ventricular pressures, $p_{v}^{L}\left(t\right)$ and $p_{v}^{R}\left(t\right)$ following (Avanzolini et al., 1988):

(14) $$p_{v}^{L}(t)=\left\{ \begin{array}{l} p_{0}^{L}\sigma (t)+E_{v}^{L}(V^{L}-V_{0}^{L})+R_{v}^{L}\dot{V^{L}}\;0<t<t_{s}\;(\text{systole})\\ E_{d}^{L}(V^{L}-V_{0}^{L})\;t_{s}\leq t<t_{c}\;(\text{diastole}), \end{array}\right.$$

where $p_{0}^{L}$ is the peak isovolumetric pressure at the volume $V_{0}^{L}$, $E_{v}^{L}=E_{d}^{L}+E_{s}^{L}\sigma \left(t\right)$ is the time-varying elastance, *E*_*d*_ is the diastolic passive elastance, $E_{s}^{L}$ is the systolic passive elastance, the activation function σ(*t*) = [1−cos(2π*t*/*t*_*s*_)] during systole and 0 during diastole, *t*_*s*_ = 0.4s is the time at end of systole, *V* is the ventricular volume, $\dot{V^{L}}$ is the rate of volume change, and $R_{v}^{L}$ is the resistance of the ventricular myocardium. A similar expression holds for $p_{v}^{R}$.

The reference pressure *p*_0_ in (5) is set to the time-averaged value of the left or right ventricular pressure for the left and right coronary arteries, respectively (Mynard et al., 2014). The large coronary arteries in this model mostly lie outside the myocardium. The terminal large arteries each connect to a vascular bed of smaller vessels represented by a structured tree. The contraction of the myocardium during systole squeezes the vascular bed and thus increases the pressure at the end of each terminal artery, where it meets its vascular bed. To account for the effects of the myocardial contraction on the coronary vessels, we introduce a time-dependent feedback pressure *p*_*f*_(*x, t*) that is added to the distal boundary conditions for each terminal large artery, i.e., where each terminal vessel is connected to its vascular bed (represented by a structured tree); this increases the resistance to flow due to the compression of the vascular beds during systole (Mynard, 2011). We choose *p*_*f*_ to be proportional to ventricular pressure plus the coronary bed pressure *p*_*s*_ (Mynard and Smolich, 2016), i.e.,

(15) $$\begin{array}{l} p_{f}\left(x,t\right)=\varphi p_{v}\left(t\right)+p_{s}, \end{array}$$

where *p*_*s*_ = 20mmHg (Hoffman and Spaan, 1990). The ventricular-pressure feedback ratio φ depends on the location of the vascular bed supplied by the terminal vessel because the left and right ventricle peak-systolic pressures are different; see Table 2. The chosen values give physiologically realistic pressure profiles.

**Table 2:** The ventricular-pressure feedback ratio is applied to all *terminal* coronary vessels, and depends on the location of the vascular beds within the myocardium. The values are chosen so that the resulting pressure profile is physiological.

**Supply location**	**φ**	**Vessel branches**
Left ventricle	33%	PDA, LAD3, LAD4, DIAG,
		MARG1, MARG2, MARG3
Right ventricle	25%	PLA
Septum	22%	LAD1,LCX3

### 2.5. The WI Analysis

The net WI is a synthesis of different waves, for example the WI may consist of a large forward wave minus a smaller backward one. The primary forward wave is caused by ventricular contraction and transmitted via the aorta into the coronary tree. Backward propagating waves are caused by reflections at bifurcations and also by myocardial contraction in the vascular beds.

To perform the WI analysis, the forward and backward wave fronts of the pressure $\delta p_{i}^{\pm }$ and velocity $\delta u_{i}^{\pm }$ are defined as in Qureshi and Hill (2015):

(16) $$\begin{array}{l} \delta p_{i}^{\pm }=0.5\left(\delta p_{i}\pm \rho c_{p_{i}}\delta u_{i}\right),\;\delta u_{i}^{\pm }=0.5\left(\delta u_{i}\pm \frac{\delta p_{i}}{\rho c_{p_{i}}}\right), \end{array}$$

over the sample times *t*_*i*_ = *iδt* ∀*i* ∈ [0, *N*], *t*_*i*_ ∈ [0, *T*], where $c_{p}=\sqrt{\frac{A}{\rho }\frac{\partial p}{\partial A}}$ is the computed pulse wave velocity in the artery. The wave intensity is then (Qureshi and Hill, 2015)

(17) $$\begin{array}{l} W_{i}^{I\pm }=\frac{\delta p_{i}^{\pm }}{\delta t}\frac{\delta u_{i}^{\pm }}{\delta t}. \end{array}$$

Waves are assumed to be linearly composed, and the net effect of the waves can be studied by analyzing the forward compression and decompression waves (FCW, FDW), and the backward compression and decompression waves (BCW, BDW).

The physiological events that determine the WI patterns of the coronary arteries can be distinguished by six phases (Davies et al., 2006): (1) During early systole, the coronary WI pattern begins with a manifest BCW and decreasing LCX velocity. This BCW is generated by myocardial contraction, which compresses the small vessel beds (Chilian and Marcus, 1982). (2) The systolic FCW caused by contraction of the left ventricle moves from the aorta to the coronary vessels. (3) Later in systole, a secondary BCW is probably generated by reflection sites such as distal coronary artery pulsation or vessel branches. (4) In proto-diastole, the myocardium begins to relax and generates an FDW which lowers the pressures in the coronary arteries. (5) Concomitantly, a dominant BDW is thought to result from ventricular relaxation; the small vessels are decompressed, which results in lower pressure at the vascular bed and a lower resistance of the micro-circulation. (6) At the end of diastole, it is speculated that a late FCW is generated from the proximal end of the coronary arteries due to the closure of the heart valve. In identifying these wave components, WI has proved to be an efficient diagnostic tool.

## 3. Results

### 3.1. The Parameters

All the parameters and their values used in the model are listed in Table 3 along with the corresponding references.

**Table 3:** The parameters used in the model.

**References and equations**	**Symbols **&** values**	**Symbols **&** values**
Olufsen (1999); Olufsen et al. (2000)	γ = 0.41	*η* = 1.16
(1)–(2)	*ξ* = 2.76	*r*_*m*_ = 10 μm
	α = 0.9	β = 0.6
	*l*_*rr*_ = 25	
Olufsen (1999); Olufsen et al. (2000)	ρ = 1.055 g *cm*^−3^	μ = 0.0047 g s^-1 cm^-1
(7)–(13)	*k*_1_ = 2 × 10^7^ g s^-2 cm^-2	*k*_2_ = -22.5 cm^-1
	*k*_3_ = 8.65 × 10^5^ g s^-2 cm^-1	
Avanzolini et al. (1988)	Left ventricle	Right ventricle
(14)	$R_{v}^{L}$ = 0.08 mmHg s ml^−1^	$R_{v}^{R}$ = 0.0175 mmHg s ml^−1^
	$E_{s}^{L}$ = 1.375 mmHg s ml^−1^	$E_{s}^{R}$ = 0.3288 mmHg s ml^−1^
	$E_{d}^{L}$ = 0.1 mmHg s ml^−1^	$E_{d}^{R}$ = 0.03 mmHg s ml^−1^
	$V_{0}^{L}$ = 11.29 ml	$V_{0}^{R}$ = 3.33 ml
	$p_{0}^{L}$ = 50 mmHg	$p_{0}^{R}$ = 24 mmHg

### 3.2. The Baseline Coronary Flow and Pressure

**Figure 2:**
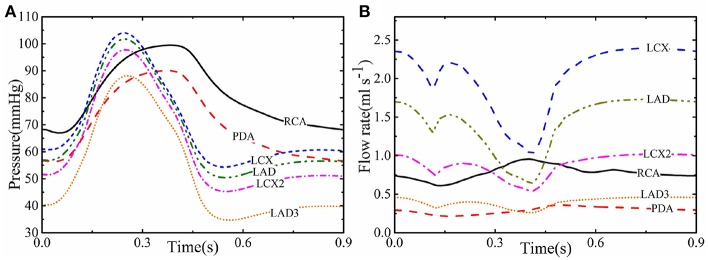
The simulated pressure **(A)** and flow rate **(B)** for different coronary vessels.

Figures 2A,B show the computed profiles of the pressure and flow rate in different coronary vessels throughout the cardiac cycle. At the beginning of systole, despite the rapid increase in the pressure, there is a sharp dip in flow-rate curve of most of the vessels; this is caused by the active heart contraction. The flow rate then increases with the pressure until it peaks, then both drop at the end of systole at about 0.4 s. In diastole, although pressure remains low, the flow rate increases in most of the coronary vessels due to the myocardial relaxation and *p*_*f*_(*t*) drops. However, the flow rate in the smaller vessels of the coronary trees, such as LDA3, remains low at 0.5 *ml s*^−1^, presumably because they are less affected by the myocardial relaxation. In the right coronary vessels (e.g., RCA) the flow rate has a different trend as the pressures and contraction are lower in the right ventricle than in the left ventricle.

### 3.3. Comparison Between Experiments and Published Results

**Figure 3:**
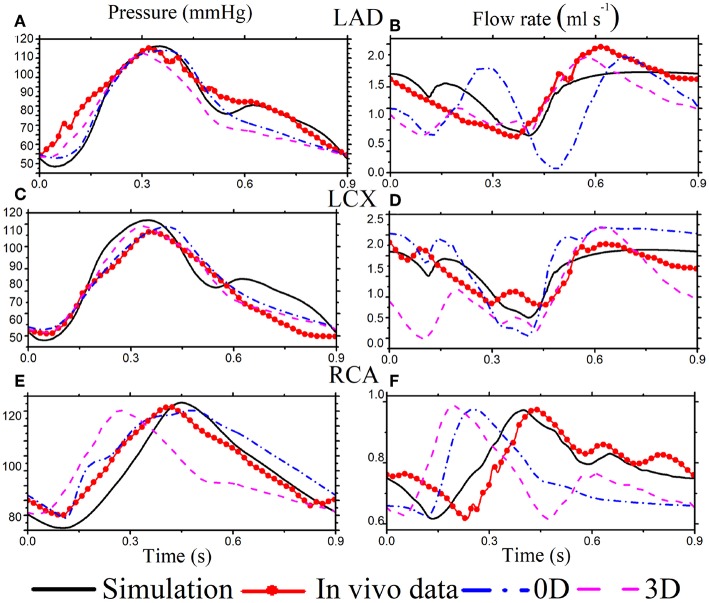
Simulation results (black solid) for selected coronary vessels [The pressure in LAD **(A)**, LCX **(C)**, and RCA **(E)**. The flow rate in LAD **(B)**, LCX **(D)**, and RCA **(F)**] are compared with published *in vivo* data (red solid dots), the corresponding 0D model (dashed dot blue), and a 3D model (dashed pink).

The simulated pressure and flow waveforms in the main vessels of coronary tree are compared with our own *in vivo* measurements in the LCX, as well as *in vivo* experiments (Duncker and Merkus, 2004; Spaan, 2012; Pijls and De Bruyne, 2013) and 0D (Duanmu et al., 2018), and a 3D Kim et al. (2010) waveform from healthy adults (Figure 3). The *in vivo* flows in LAD and RCA are from Figure 3 of Duncker and Merkus (2004), and the pressures in LAD and RCA are separately from Figure 5.13 and Figure 6.10 in Pijls and De Bruyne (2013). The cardiac cycles of the clinical data are adjusted to have the same heart beat of 0.9 s. The simulated flow rate agrees well with our own experimental data in the LCX. Although the geometries of the coronary structure are different, our model also compares reasonably well with the published experimental measurements.

The flow profiles from our 1D simulated model are different from those of the 0D (Duanmu et al., 2018) and 3D (Kim et al., 2010) models in some vessels. As shown in Figure 3D, the 0D flow rate of LAD is different during systole, which might be because of the lumped parameter assumption. In right coronary vessel RCA, the flow rate from the 0D and 3D models becomes systolic dominant, which might result from the 0D outlet boundary condition, and is not consistent with the experimental data in Figure 3F. Overall, our model compares reasonably well with the published experimental measurements (Duncker and Merkus, 2004; Spaan, 2012; Pijls and De Bruyne, 2013).

Diastolic dominant coronary arterial flow is also captured in our model. It is caused by decompression of the terminal bed consequent to myocardial relaxation (Van der Horst et al., 2013), which compresses the terminal vascular bed. This myocardial contraction's effect on the coronary flow is incorporated in the model using the feedback pressure. The flow in the right coronary vessel is less dominant in diastole, which is due to the lower blood supply to the ventricle from RCA.

### 3.4. Wavefronts and WI Analysis

**Figure 4:**
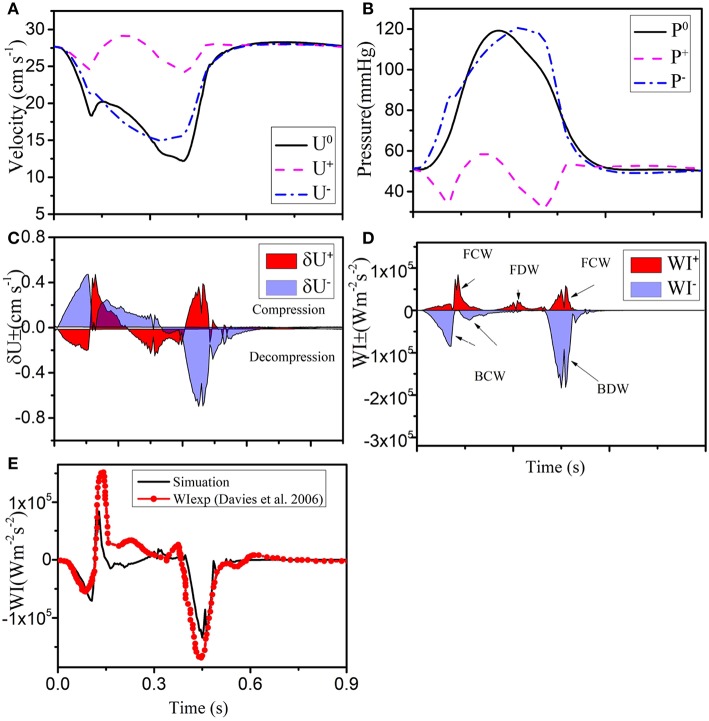
**(A)** Simulated velocity (*U*^0^) and **(B)** pressure (*P*^0^) waveforms (solid black), decomposed into forward (“+” dashed pink), and backward (“-” dashed-dot blue) running components at the midpoint in the LCX. The separated wave fronts and intensities are shown in **(C)** $\delta U_{i}^{+}$ and **(D)** $\delta WI_{i}^{+}$ (shaded red), $\delta U_{i}^{-}$ and $\delta WI_{i}^{-}$ (shaded blue). **(E)** The total simulated wave intensity (black line) is also compared with the experimental data (red line with dots) of Davies et al. (2006).

We now apply the wave intensity analysis to identify the forward and backward traveling waves. Since most of the published *in vivo* measurements and modeling results are for the LCX vessel, we use the results for LCX as a benchmark case. In Figures 4A,B, forward and backward velocity and pressure components are calculated from the pressure and velocity waveforms. The WI results for the LCX are shown in Figure 4C, where positive values indicate compression and the negative ones decompression; $\delta U_{i}^{+}$ is the forward flow and $\delta U_{i}^{-}$ is backward. Six major WI components can be identified in Figure 4D, which are consistent with previous studies (Davies et al., 2006). Figure 4E shows that our results are comparable with the measurements by Davies et al. (2006).

**Figure 5:**
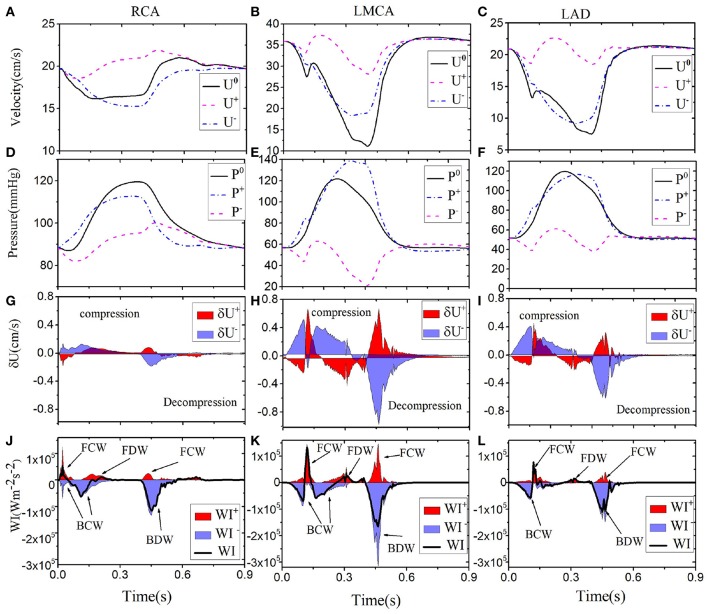
Simulated waveforms for different coronary vessels; the right coronary vessel RCA (1st column): velocity **(A)**, pressure **(D)**, wave front **(G)**, and intensities **(J)**. LMCA (2nd column): velocity **(B)**, pressure **(E)**, wave front **(H)**, and intensities **(K)**. LAD (3rd column): velocity **(C)**, pressure **(F)**, wave front **(I)**, and intensities **(L)**. Simulated velocity (*U*^0^) and pressure (*P*^0^) waveforms (solid black), decomposed into forward (“+” dashed pink) and backward (“-” dashed-dot blue) running components at the midpoint in each vessel. The separated wave fronts and intensities, $\delta U_{i}^{+}$ and $\delta WI_{i}^{+}$ (shaded red), $\delta U_{i}^{-}$ and $\delta WI_{i}^{-}$ (shaded blue), are displayed at the bottom.

Next, we show our results at three different locations of the coronary tree (RCA, LCD, LMCA) in Figure 5. The blood pressure in the RCA (Figure 5D) is almost the same as the pressure in the LCX (Figure 4), but the flow in the RCA (max 22.5 cm s^−1^) is lower. The six different waves in the RCA are shown in Figure 5J, the WI are much less than those in the LCX, which is also reflected by the less flow fluctuation in the RCA. In the LMCA, the flow velocity increases with a maximum value of 36 cm s^−1^ (Figure 5B), while the pressure is similar to those in the LAD and LCX, as shown in Figure 5E. The increased flow velocity in the LMCA also corresponds to the increased WI compared to the values in the LCX, shown in Figure 5K. The flow, pressure and WI in the LAD are very close to the LCX flow and pressure, as shown in Figure 5.

The area enclosed by each of the major waves in one heart beat represents the flow energy. The wave energy analysis helps to estimate the physiological reaction of heart failure if coupled with other information such as coronary flow or pressure (Kyriacou et al., 2012; Lee et al., 2016). The flow energy from our model is compared with experimental studies from Davies et al. (2006) and Lee et al. (2016) in Table 4. The waves (a), (b) and (c) in Table 4 are due to systole, and there is considerable variation between all sources, probably due to differences in the measurement location and vasculature structure (Lee et al., 2016). The dominant wave BDW (e) contains the greatest energy ration, which agrees well with the two experimental studies. The FDW is due to reflection at junctions between vessels.

**Table 4:** The comparison of cumulative proportional wave intensity.

**Wave type**	**Experiment by Davies et al. (2006)%**	**data from Lee et al. (2016)%**	**Our model %**
(a)BCW	1.9 ± 2.1	5.1	19.8
(b)FCW	22.3 ± 7.9	27.4	12.8
(c)BCW	20.5 ± 2.9	2.8	11.6
(d)FDW	18.9 ± 4.0	13.4	5.6
(e)BDW	30 ± 5.7	37.3	45.4
(f)FCW	6.1 ± 2.4	14.1	7.5

### 3.5. Pathological Cases

We now compare the baseline results with the pathological cases. Four different pathological cases are considered. (A) Stiffer large arteries which are associated with a higher risk of cardiovascular diseases such as stroke and coronary atherosclerosis (Weber et al., 2004). This is modeled by increasing *k*_3_ by 100% in Equation (6). (B) Rarefaction (reduced vessel density) in the terminal vascular bed is modeled by reducing the exponent *ξ* from 2.76 to 2.4 in (1), corresponding to a 20% reduction in small vessels (Olufsen et al., 2012). In borderline hypertension and normal young offspring of parents with hypertension, it has been suggested that micro-circulatory rarefaction may be a cause of hypertension (Olufsen et al., 2012). (C) Changed feedback pressure is associated with heart pump function and can be indicative of several coronary epicardial diseases (Siebes et al., 2009). This is considered by varying φ from 0 to 60% for LAD1; in particular, when φ=22%, LAD1 is connected to septum (as shown by our CT scan), and when φ=33%, LAD1 is connected to the left ventricle which sometimes occurs (Mynard and Smolich, 2016). (D) Variation in coronary geometry can have an impact on the model results; the changes could be pathological or due to inaccurate measurements (Uus et al., 2015). This is assessed by shrinking or enlarging the arterial length and radius by ± 5 and ±10%, respectively.

**Figure 6:**
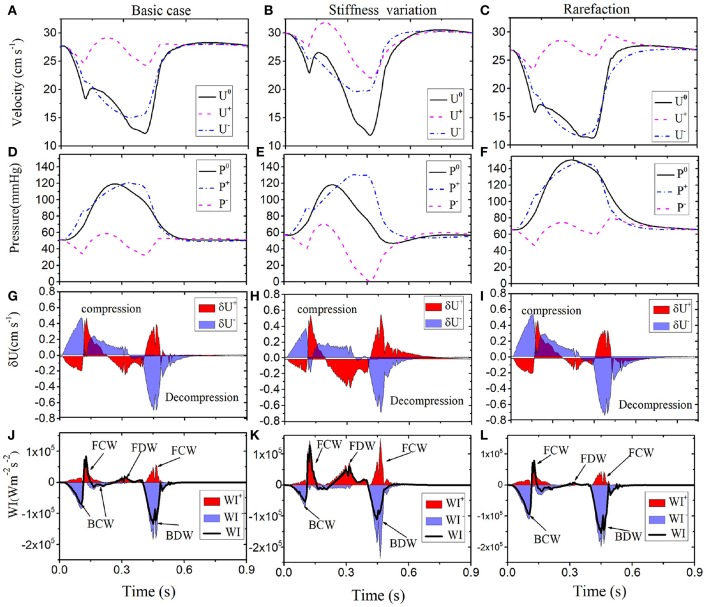
Comparison of results along LCX are shown for the Baseline (left): velocity **(A)**, pressure **(D)**, wave front **(G)**, and intensities **(J)**. Case A (middle): velocity **(B)**, pressure **(E)**, wave front **(H)**, and intensities **(K)**. Case B (right): velocity **(C)**, pressure **(F)**, wave front **(I)**, and intensities **(L)**. Simulated velocity (*U*^0^) and pressure (*P*^0^) waveforms (solid black), decomposed into forward (“+” dashed pink) and backward (“-” dashed-dot blue) running components at the midpoint in the LCX of different conditions (1st and 2nd row). The separated wave fronts and intensities, $\delta U_{i}^{+}$ and $\delta WI_{i}^{+}$ (shaded red), $\delta U_{i}^{-}$ and $\delta WI_{i}^{-}$ (shaded blue), are displayed in the 3rd and 4th rows. The black line in the bottom panel is the total sum of the WI.

The comparison with cases A and B for the LCX are shown in Figure 6. We can see that a higher stiffness in Case A results in an increased velocity (30–35 cm s^−1^ peak), but pressure changes are moderate. In Case B, pressure is significantly increased (from the peak value of 120 to 150 mmHg), but velocity changes are not significant. It is interesting to see more information is provided by WI analysis. In particular, the difference between the Baseline and Case B is smaller in both pressure and velocity waves, but more changes are shown in Case A.

**Figure 7:**
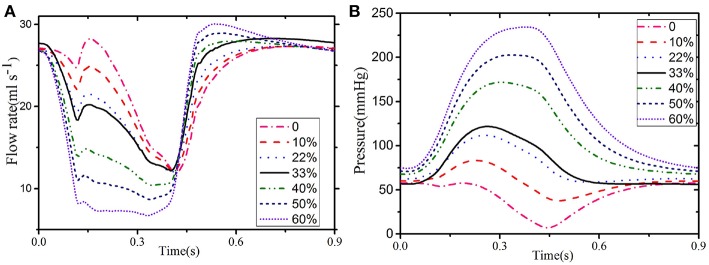
Flow **(A)** and pressure **(B)** in the LCX for different values of φ: 33% when the LCX is connected to the ventricular and 22% when connected to the septum.

Figures 7A,B show the comparison of the results for LCX between the Baseline (φ = 0.33) and Case C (φ = 0.0−0.6). Clearly, the value of φ has a great impact on the simulated results. Figure 7 shows that the coronary flow increases as φ decreases during systole, and vice versa during diastole. The dynamic feedback pressure is systolic dominant, and the blood flow increases when the feedback pressure is lower.

Finally, we compare the baseline with Case D, where the overall size of the coronary tree (including all the radii and lengths) is changed to (90, 95, 105, and 110%) of the baseline case. We observed that although the wave profiles are similar in all cases (not shown), the magnitudes are changed, as shown in Table 5. We first note that flow resistance is approximately proportional to vessel length. We now explain the influence of size on WI. The arterial stiffness (elastic modulus) is the inverse of compliance, and a stiffer vessel has a higher FDW (Davies et al., 2006). The decrease in size is equivalent to a compliance reduction which results in augmentation of stiffness. Hence, the increase/decrease of the geometrical measurement corresponds to a low/high FDW.

**Table 5:** Effects of LCX size changes. i.e., changes in length and distal radius. The maximum flow, pressure and WI are given as the percentages of the corresponding baseline values.

**Size percentage %**	**Flow peak %**	**pressure peak %**	**WI peak %**	**FDW peak %**
90	118	120	105	340
95	108	109	90	142
105	92	93	82	47
110	84	88	111	16

## 4. Discussion

In this study, the systemic arteries model originally developed by Olufsen (1999) has been extended to simulate the dynamics of the anatomically based coronary arteries. Although a number of other multiscale simulations of coronary flow have been carried out (Mantero et al., 1992; Mynard and Smolich, 2015), none of these have considered a structured-tree model as in Olufsen (1999) and incorporate myocardial contraction as feedback pressure in the vascular network. The pattern of WI in coronary arteries is quite different from the systemic circulation due to myocardial contraction, and the net WI of the LCX agrees well with experimental data, as shown in Figure 4E.

The simulated waveforms demonstrate that the flow velocity and WI patterns differ between the left and right coronary vessels. The shapes of the flow, pressure and wave intensity curves in the left coronary vessels (e.g., columns 2 and 3 in Figure 5) are the similar, but with different amplitudes. The greatest flow occurs during diastole. Larger flow and WI in the LMCA can be observed during diastole since it is close to the aortic root. Compared with left coronary vessels, the WI in the RCA is different and much smaller. This is because there are fewer structural branches and lower terminal pressure differences (Hadjiloizou et al., 2008) in the right coronaries.

We also model some pathological cases and show that a higher flow is always associated with an increase in the three forward wavefronts in systole. Our results also confirm that increased stiffness of the coronary arteries will increase the FDW during diastole, consistent with observations by Davies et al. (2006). The stiffened arteries can create an energy surge into coronary vessels. Hence, the FDW might be a good indicator for diagnosis of diseased coronary arteries.

The augmentation of pressure can be seen in rarefaction even though there is no obvious change in the flow. However, rarefaction contributes to hypertension with the increased BCW and BDW. This abnormally high pressure is transmitted into the micro-vascular networks, which can lead to pressure imbalance and vessel destruction (Feihl et al., 2009). Therefore, the BCW and BDW in the WI analysis can help to predict the loss of small vessels in the vascular beds (territorial ischemia). Although the mechanism of rarefaction-induced hypertension is complex (Feihl et al., 2009), our model analysis may provide a quantitative link between vasculature reduction and WI.

Finally, we study the effects of change in geometry in our model. Our results show that the FDW is very sensitive to geometry variation, which can have a 3-fold increase if the size is reduced by 10%, and 5-fold if the size is increased by 10%. This suggests that the FDW can be a good indicator of size change. However, we should also treat it with caution, as small errors in geometry measurements may induce large errors in the FDW. The other quantities, however, are less sensitive to size perturbations.

We now mention the limitations of our approach. The 1D model is based on a simplified tapered geometry for a 3D *in vivo* coronary tree. The complicated biophysical interaction between the ventricle and coronary flow is modeled using a dynamic space-dependent feedback pressure on the large vessels. In addition, we have not considered dynamic feedback pressure within the structured-tree model, which will change the impedance of the terminal vessels. Moreover, the venous pressure and flow were neglected in our model.

In summary, a 1D model based on the CT scans of coronary circulation is developed. The model employs a structured-tree model for the coronary vascular beds and includes combined feedback pressure resulting from contraction of the heart wall as an additional term in the boundary condition at the linking terminal arteries to the vascular beds. Our model agrees well with previous published studies and data. In addition, we use WI analysis to quantify flow and pressure waves in the coronary arteries. Pathological conditions such as stiffened coronary arteries and vascular rarefaction, as well as changes in geometry and the ventricular-pressure feedback ratio, are also studied.

## Data Availability

The dataset for this manuscript is not publicly available because it is limited to clinical and research use in The Second Affiliated Hospital of Nanjing Medical University. Requests to access the datasets should be directed to ZD.

## Author Contributions

ZD conceptualized and designed the study. The need for a structured-tree model for the coronary vasculature was identified by XL and NH. NH provided the code that was modified by WC and HG. XY helped with measuring the clinical experiment and collecting data. XL and NH helped with the analysis and interpretation of the data, critically revising the manuscript, and adding important intellectual content. All authors gave approval for the final version of this manuscript to be published and agreed to be accountable for all aspects of the work.

### Conflict of Interest Statement

The authors declare that the research was conducted in the absence of any commercial or financial relationships that could be construed as a potential conflict of interest.
